# Effect of Astaxanthin on Human Sperm Capacitation

**DOI:** 10.3390/md11061909

**Published:** 2013-06-03

**Authors:** Gabriella Donà, Ivana Kožuh, Anna Maria Brunati, Alessandra Andrisani, Guido Ambrosini, Guglielmo Bonanni, Eugenio Ragazzi, Decio Armanini, Giulio Clari, Luciana Bordin

**Affiliations:** 1 Department of Molecular Medicine-Biological Chemistry, University of Padova, Padova 35131, Italy; E-Mails: gabriella.dona@unipd.it (G.D.); kozuh.ivana@gmail.com (I.K.); annamaria.brunati@unipd.it (A.M.B.); giulio.clari@unipd.it (G.C.); 2 Department of Women’s and Chilren’s Health, University of Padova, Padova 35131, Italy; E-Mails: alessandra.andrisani@unipd.it (A.A.); guido.ambrosini@unipd.it (G.A.); 3 Department of Medicine—Endocrinology, University of Padova, Padova 35131, Italy; E-Mails: guglielmo.bonanni@unipd.it (G.B.); decio.armanini@unipd.it (D.A.); 4 Department of Pharmaceutical and Pharmacological Sciences, University of Padova, Padova 35131, Italy; E-Mail: eugenio.ragazzi@unipd.it

**Keywords:** human sperm, astaxanthin, Tyr-phosphorylation, acrosome reaction

## Abstract

In order to be able to fertilize oocytes, human sperm must undergo a series of morphological and structural alterations, known as capacitation. It has been shown that the production of endogenous sperm reactive oxygen species (ROS) plays a key role in causing cells to undergo a massive acrosome reaction (AR). Astaxanthin (Asta), a photo-protective red pigment belonging to the carotenoid family, is recognized as having anti-oxidant, anti-cancer, anti-diabetic and anti-inflammatory properties and is present in many dietary supplements. This study evaluates the effect of Asta in a capacitating buffer which induces low ROS production and low percentages of acrosome-reacted cells (ARC). Sperm cells were incubated in the presence or absence of increasing concentrations of Asta or diamide (Diam) and analyzed for their ROS production, Tyr-phosphorylation (Tyr-P) pattern and percentages of ARC and non-viable cells (NVC). Results show that Asta ameliorated both sperm head Tyr-P and ARC values without affecting the ROS generation curve, whereas Diam succeeded in enhancing the Tyr-P level but only of the flagellum without increasing ARC values. It is suggested that Asta can be inserted in the membrane and therefore create capacitation-like membrane alteration which allow Tyr-P of the head. Once this has occurred, AR can take place and involves a higher numbers of cells.

## 1. Introduction

Capacitation is a set of alterations leading to the acrosome reaction (AR), an exocytotic process by means of which hydrolytic enzymes (e.g., acrosin) are released to allow sperm to fertilize oocytes [1]. Augmented membrane fluidity due to lipid modifications, Ca^2+^ influx, generation of controlled amounts of reactive oxygen species (ROS) and phosphorylation of proteins on serine (Ser), threonine (Thr) and tyrosine (Tyr) residues [2,3,4,5] are the main modifications which sperm must face in order to be correctly capacitated and prompt in undergoing AR. Defective antioxidant defense may be responsible for the increase in oxidative stress (OS) observed in the seminal fluid of infertile patients [6], as evidenced by their lower total antioxidant capacity (TAC) [7]. ROS may also be generated by sperm itself, and enhanced ROS production has been observed in infertile men [8] and correlated with the percentages of both apoptotic and necrotic sperm [8,9,10].

In recent reports, we showed that human sperm endogenous ROS production is essential for correct capacitation, evaluated as the percentage of acrosome-reacted cells (ARC) directly dependent on Tyr-phosphorylation (Tyr-P) of the sperm head [11]. A classification score was also defined based on endogenous ROS production, for better characterization of sperm samples [12]. Optimal capacitation was observed only in sperm which expressed endogenous ROS within the correct range of cut-off values for normal sperm, allowing the largest number of cells to achieve head Tyr-P and AR [12].

Synthesized by the microalgae *Haematococcus pluvialis* [13], astaxanthin (Asta) is a photo-protective red pigment belonging to the carotenoid family and sharing a similar beta-carotene structure [14]. Its molecule consists of 40 carbon atoms, divided into a central portion containing 22 carbon atoms linked with 13 conjugated double bonds and two terminal benzene rings containing hydroxyl and ketone groups, giving rise to the higher polar structure of Asta compared with other carotenoids [15].

Known for its antioxidant activity, Asta is also recognized to have anti-cancer [16], anti-diabetic and anti-inflammatory [17] properties. It is present in many dietary supplements and is used for sperm quality improvement treatment. In seminal fluid it significantly decreases ROS production and has positive effects on some semen parameters and male fertility by increasing sperm concentration and linear velocity, thus ameliorating fertilizing power [18]. The aim of this *in vitro* study was to evaluate the effect of Asta on human sperm endogenous ROS production, Tyr-P and AR.

## 2. Results and Discussion

### 2.1. Effect of Asta on Sperm Endogenous ROS Generation

Samples were incubated with increasing concentrations of Asta. An aliquot from each sample was immediately withdrawn to be analyzed for ROS generation in a luminometer for 180 min, with luminol as luminescent source.

**Figure 1:**
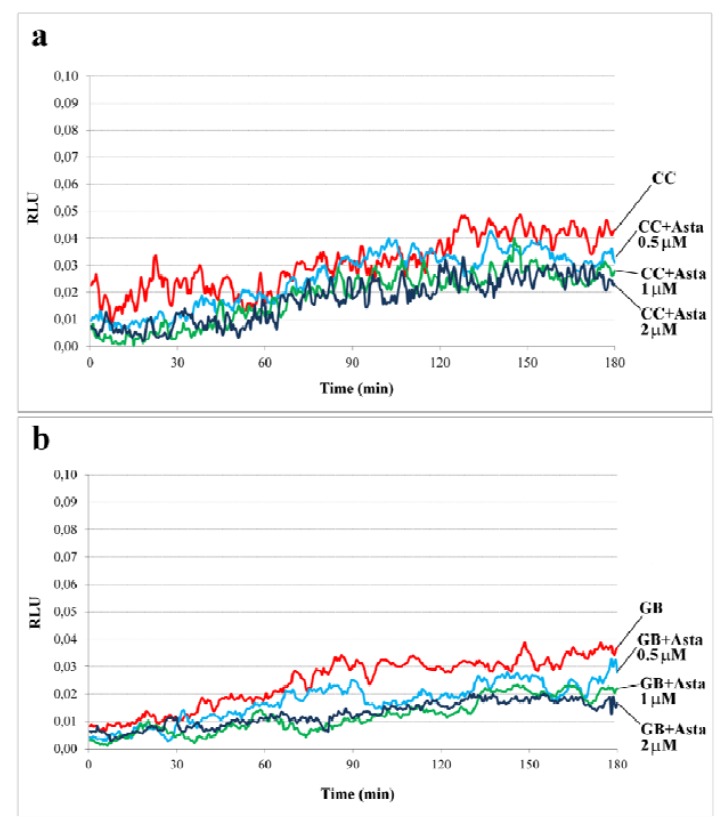
Time-dependent and redox effects on endogenous ROS generation of human sperm during capacitation. (**a**) in presence of sperm; (**b**) incubation media (Gamete Buffer, GB) as background.

Figure 1 shows the curve of ROS generation (pattern a). Asta did not induce any appreciable variation in ROS generation, mainly H_2_O_2_ content, either in the presence of sperm or in the incubation media as background (pattern b).

Sperm from two volunteers for each experiment (*n* = 6 separate experiments) was collected to form a pool with a sufficient number of cells. Sperm was incubated for up to 180 min in capacitating conditions in the absence (CC) or presence of increasing concentrations of Asta (0.5, 1, 2 μM). Luminol chemiluminescence was monitored during sperm capacitation. Results are expressed as moving averages of Relative Luminescence Units (RLU)/30 s for 2 × 10^6^ cells. Background luminescence of sample buffer (GB) was assessed and reported. Figure 1 is representative of six separate experiments, each in triplicate.

None of these conditions allowed the cells to achieve the correct endogenous ROS content since all samples remained under the threshold of 0.05 RLU [12]. It is hypothesized that Asta stimulates intracellular oxygen metabolites such as superoxide, which cannot readily escape the plasma membrane and interact with luminol, with the risk of underestimating the effective superoxide concentration. However, the peculiarity of Asta molecule is its series of conjugated double bonds which can remove free radicals by delocalizing their electronic energy via the carbon–carbon chain. This gives Asta a considerable antioxidant potential, since it is neither destroyed nor does it become a pro-oxidant in the process [19], as evidenced in cultured cells in which Asta has been demonstrated to inhibit intracellular radical generation [20].

At the end of incubation, aliquots of cells were withdrawn from all samples, to be analyzed for functioning, *i.e.*, their ability to undergo Tyr-P and AR.

### 2.2. Effect of Asta on Sperm Tyr-P Pattern

**Figure 2:**
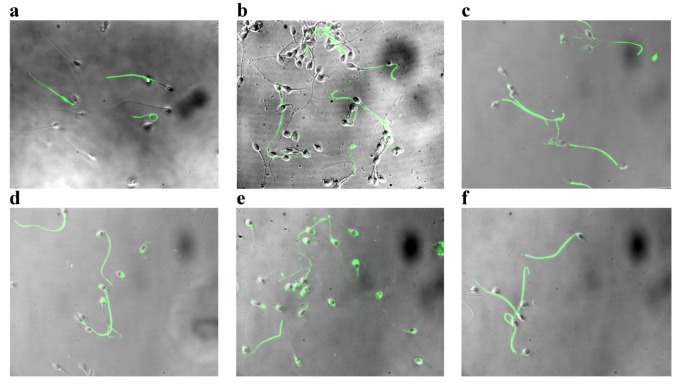
Tyr-P detection by immunofluorescence cytochemistry of sperm incubated in capacitating conditions (**a**) for 0 min (*T*_0_); (**b**) 180 min in absence (capacitating conditions (CC)), and presence of (**c**) 0.5, (**d**) 1, (**e**) 2 μM Asta, or (**f**) 1 μM Diam.

**Figure 3:**
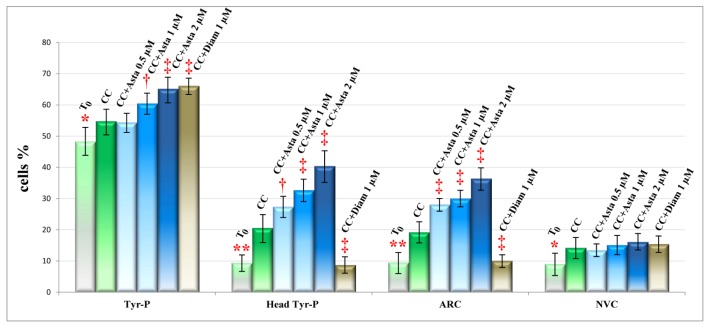
Sperm cells, at *T*_0_ or incubated for 180 min in capacitating conditions with increasing concentrations of Asta, or 1 μM Diam, were analyzed for Tyr-P pattern, acrosome-reacted cells (ARC) and viability (non-viable cells (NVC)) by immunofluorescence cytochemistry (see Methods). Number of cells expressed as % of total number of cells showing Tyr-P in any part of cell body or in head, were detected and reported as Tyr-P cells and Tyr-P head, respectively. Percentages of cells undergoing acrosome reaction (AR) or NVC were also reported. Values are expressed as means ± SD. † *p* < 0.01; ‡ *p* < 0.0001 comparison between various concentrations of Asta or Diam *vs.* CC as reference; Student’s *t*-test for paired data. * p < 0.01; ** p < 0.0001 comparison CC *vs. T*_0_; Student’s *t*-test for paired data.

The Tyr-P pattern (Figure 2) was evaluated at *T*_0_, *i.e.*, at the beginning of incubation, and after 180 min in the presence of increasing concentrations of Asta. In sample *T*_0_, Tyr-P was located mainly in the mid-piece region and tails of sperm (Figure 2, pattern a), but involved only a low percentage of cells. After 180 min in capacitating conditions (CC) (pattern b), only a few cells presented Tyr-P in the head region, as expected on the basis of ROS generation values expressed by cells (Figure 1a). Interestingly, the addition of Asta did manage to trigger sperm head Tyr-P in a dose-dependent manner (0.5 < 1 < 2 μM; Figure 2c,d,e, respectively) involving a significantly augmented number of Tyr-P cells although ROS generation content was not affected at all (Figure 1a). In order to evaluate whether Asta acts by either directly affecting the Tyr-P process or altering Tyr-phosphorylatable substrates, further sperm aliquots were incubated with diamide (Diam), a mild oxidant known to inhibit Tyr-protein phosphatase activity by oxidizing the cysteine residues of their catalytic domain [21]. Diam induced high Tyr-P level as shown by the enhanced Tyr-P labeling of sperm (pattern f), without modifying the ROS generation curve (not shown) (for quantitative data and statistics, see Figure 3).

### 2.3. Effect of Asta on Sperm Viability and Acrosome Reaction (AR)

At the end of incubation, aliquots of sperm suspensions were analyzed for percentages of non-viable cells (NVC) and ARC. Cells were counted and values are shown in Figure 3, together with the percentage of cells presenting Tyr-P.

The percentage of Tyr-P cells was significantly augmented in the presence of Asta in a dose-dependent way (65.2 ± 3.9% with 2 μM Asta *vs.* 54.8 ± 4.0% CC; *p* < 0.0001) but, more interestingly, the percentage of cells presenting Tyr-P in the head region was much more increased by Asta, reaching 27.4 ± 3.4% at 0.5 μM (*p* < 0.01), 32.7 ± 3.7% at 1 μM (*p* < 0.0001) and 40.4 ± 4.6% at 2 μM Asta (*p* < 0.0001) *vs.* 20.6 ± 4.2% CC. This was followed by a related increase in the percentage of AR cells which, at 2 μM Asta, were almost twice as many as CC (36.4 ± 3.6% *vs.* 19.2 ± 3.3%, *p* < 0.0001). Interestingly, Diam treatment induced as high a percentage of Tyr-P cell (66.1 ± 2.4%) as did Asta 2 μM, but the percentage of cells with Tyr-P in the head was by far the lowest (8.7 ± 2.5%) compared with both Asta and CC samples, and resembled that of sample *T*_0_. Concomitantly, the ARC percentage of Diam samples (10.1 ± 2.2%) was also similar to that at *T*_0_.

**Figure 4:**
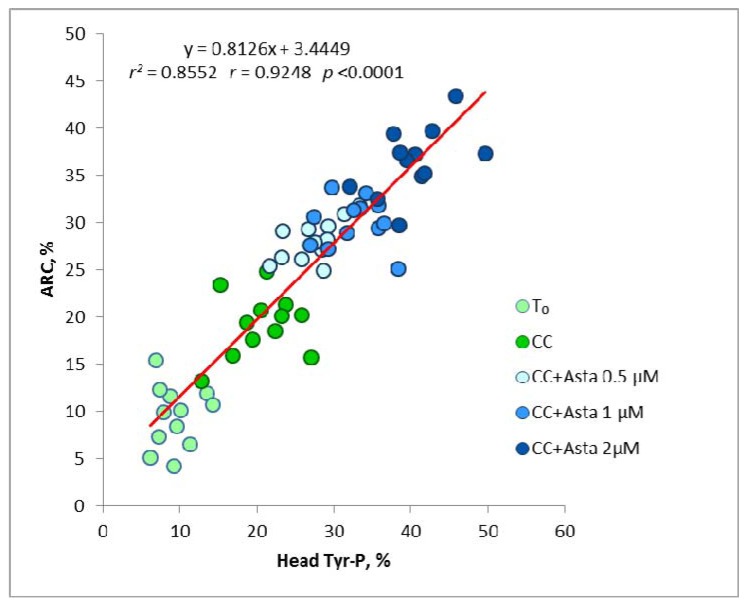
Linear regression of Tyr-P% against ARC% values for all data obtained in basal conditions and after Asta addition.

As shown in Figure 4, a close linear relationship (*r* = 0.9248, *p* < 0.0001) was found between the percentages of cells presenting head Tyr-P and the amount of ARC, in all experimental conditions. This confirms what we had already found in our previous works [11,12]; in addition, cell activation, linearly linked to Tyr-P from the basal condition onward, is greatly enhanced by the progressive increase of Asta concentrations, demonstrating not only the close dependence of AR on the extent of Tyr-P, but also the possibility of pharmacologically modulating *in vitro* sperm cell activation.

No substantial alteration was found in the percentage of non-viable cells (NVC) in any of the conditions.

Previous studies had demonstrated that AR closely depends on the Tyr-P process of the sperm head and also on ROS generation of sperm [11,12]. In the absence of endogenous ROS, e.g., in sperm unable to produce them or in the presence of strong anti-oxidants, such as ascorbic acid, sperm cells undergo a kind of general impairment, which prevents not only their ability to undergo AR but also cell survival [11]. In the present study, sperm was incubated in a buffer which induced only slight ROS production, barely sufficient to achieve about 20% of AR. Asta greatly improved the AR percentage, in spite of the inability of cells to reach the correct ROS content production. In addition, no change in ROS content was observed following Asta addition, thus confirming previous data in HeLa human cells [20], in which Asta did not directly scavenge superoxides. This is probably because Asta, being extremely lipophilic, can be inserted in the bilayer, while the charged radicals are unable to cross cell membranes, so that contact with Asta becomes improbable or impossible [19]. What is noteworthy is that the pathway leading to Tyr-P of the sperm head is greatly improved by Asta in a dose-dependent way, but not by diamide. It is reasonable to assume that the insertion of Asta in the membrane mediates/mimics the correct membrane modifications occurring during the capacitation process. Unless this correct membrane arrangement occurs, the mere increase in the Tyr-P process, such as that induced by the diamide-related inhibition of Tyr-protein phosphatase activity, is absolutely insufficient for the acrosome reaction to take place. Tyr-P is, in fact, greatly increased but is only located in the flagellum, and the ARC percentage remains at the level of un-capacitated cells. Capacitation implies a marked reorganization of membrane architecture when cholesterol is extracted by extracellular proteins. Until now, it has been universally believed that the sperm plasmatic membrane contained special lipid micro-domains, also called rafts or detergent-resistant membranes (DRMs) [22], capable of remodeling and reorganizing themselves in the presence of extracellular proteins (in most cases, albumin), cholesterol-extracting molecules (e.g., b-methylcyclodextrin) [23] or bicarbonate [24]. Once capacitation has occurred, these DRMs migrate from the flagellum, where they occur extensively, to the peri-acrosomal region [22] where they are presumed to allow oocyte interactions.

One protein enriched in sperm membrane micro-domains is caveolin 1 (CAV 1) [22] which provides DRM scaffolding by homo-oligomerization in high molecular weight aggregates [25]. Many proteins sequestered within caveolae are inactive, since they are tethered by CAV-1 in the scaffold of the caveolae themselves, which may dissociate or cluster during capacitation [22]. According to this line of evidence, Asta, inserted into the membrane bilayer, may disrupt protein–protein or protein–lipid interactions, thus bypassing extracellular-mediated membrane reorganization. Asta, perturbing this organization, may set free proteins involved in the following acrosome reaction, even in the absence of additional stimuli, such as ROS lipid peroxidation.

## 3. Experimental Section

### 3.1. Semen Collection and Analysis

Twenty-four healthy male donors (age range: 20–35 years, average age: 27.8 years) were enrolled at the Department of Medicine-Endocrinology, University of Padova, Italy. After 3 days of abstinence, semen samples were collected by masturbation in a sterile container and then assessed for sperm parameters. All sperm samples used in this study were normal in terms of sperm count, motility, morphology, volume, fructose level and pH, according to the World Health Organization criteria [26]. All samples presenting any kind of contamination were discarded. This study was approved by the Ethics Committee for Research and Clinical Trials of our University, and all recruited donors gave their informed written consent and provided detailed lifestyle histories.

### 3.2. Chemicals

Anti-P-Tyr mouse monoclonal and goat anti-mouse IgG-fluorochrome fluorescein isothiocyanate (FITC) conjugate antibodies were purchased from Upstate (Becton Dickinson Italia SpA., Milan, Italy) and Santa Cruz Biotechnology (Heidelberg, Germany), respectively. Sperm Gradient kits and Cook Gamete Buffer (GB) was obtained from William A. Cook Australia Pty. Ltd. (Brisbane, QLD, Australia). Asta was supplied by FERpharma s.r.l. (Milan, Italy). 12-myristate-13-acetate phorbol ester (PMA) was purchased from Calbiochem (Nottingham, UK) and all other reagents from Sigma-Aldrich (Milan, Italy).

### 3.3. Sample Preparation

After semen analysis, ejaculated samples were laid on a discontinuous gradient (40/80%, Sperm Gradient kits) and centrifuged at 750× *g* for 20 min at room temperature. The seminal plasma and sperm from the 40% gradient interface were discarded, and the sperm cells from the bottom pellet (80% gradient) were gathered. Cells from two different volunteers were collected to obtain a pool with a sufficient number of cells to perform all evaluations (ROS, Tyr-P determination, AR status and viability) for each experiment (*n* = 6 separate experiments). Cells were washed with GB, re-analyzed for concentration, motility, viability and morphology, and collected in a single vial (stock sample) at a concentration adjusted to 80 × 10^6^ sperms/mL in GB. Stock samples were prepared in aliquots and incubated for up to 180 min in capacitating conditions, in the absence (CC) or presence of increasing concentrations of Asta from stock solutions of 100 mM dissolved in dimethyl sulphoxide (DMSO) to final concentrations of 0.5, 1 and 2 μM, respectively (CC + Asta 0.5 μM; CC + Asta 1 μM; CC + Asta 2 μM). In some experiments (*n* = 4) an additional aliquot was incubated in the presence of 1 μM Diam dissolved in GB (CC + Diam).

### 3.4. ROS Enhanced Chemiluminescence (ECL)

Production of ROS was measured by the chemiluminescence assay method with luminol (5-amino-2,3-dihydro-1,4-phthalazinedione) as probe [11,27]. Briefly, 2 μL of 25 mM luminol and 4 μL of 10 mg/mL horseradish peroxidase, both prepared in DMSO, were added to 200 μL of a sperm suspension at a concentration of 10 × 10^6^ cells/mL. ROS levels were determined by a luminometer (Fluoroskan Ascent FL, Labsystems, Helsinki, Finland) in the integrated mode for 180 min at 37 °C. Results are expressed as Relative Luminescence Units (RLU) per 2 × 10^6^ sperm cells. Lastly, 2 μL of a 10 mM *N*-formylmethionyl-leucyl-phenylalanine (FMLP) stock was added and, after a further 10 min of incubation, 4 μL of a 1 nM stock solution of PMA was added, to exclude leukocyte contamination [28]. Only samples with negative response to FLMP and PMA were processed.

### 3.5. Anti-P-Tyr Evaluation at Confocal Microscopy

Sperm aliquots (15 × 10^6^ cells) were centrifuged for 5 min at 2300× *g*, washed with PBS containing vanadate 1 mM and protease inhibitor cocktail, fixed with 2% (w/v) paraformaldehyde in PBS, and incubated overnight at 4 °C on slides pre-coated with poly-l-lysine [11]. Slides were rinsed twice with PBS, treated with 0.2% (v/v) Triton X-100 in PBS for 15 min at 4 °C, and incubated with anti-P-Tyr [diluted 1:20 in 1% bovine serum albumin (BSA) in PBS] for 1 h at 37 °C in a humid chamber. Slides were washed three times with PBS and then stained with anti-mouse IgG-FITC conjugate (diluted 1:100 in 1% BSA in PBS) for 1 h at 37 °C in a humid chamber, rinsed with PBS and mounted. Fluorescence was detected as described below. Staining without primary antibody was used as negative control.

### 3.6. Evaluation of Acrosome Reaction

Acrosome status was monitored with acrosome-specific FITC-labeled peanut (*Arachis hypogaea*) agglutinin (FITC-PNA) in conjunction with DNA-specific fluorochrome propidium iodide (PI) as a viability test [29]. Briefly, AR was induced by incubating aliquots (15 × 10^6^ cells) of each sample for a further 30 min at 37 °C, in the presence of 10 μM Ca^2+^ ionophore A23187 [11]. Control tubes contained DMSO, but not ionophore. Sperm aliquots were centrifuged for 5 min at 2300× *g*, resuspended in PBS and exposed to PI (final concentration 12 μM) for 10 min at room temperature. Sperm was washed with PBS, centrifuged, fixed with 2% (w/v) paraformaldehyde and incubated overnight at 4 °C on poly-l-lysine-treated slides. Permeabilized sperm cells, as described above, were stained with 1 mg FITC-PNA/mL for 15 min at 37 °C in the dark, washed and mounted. At least 200 cells were evaluated for each sample, and fluorescence was detected with the UltraView LCI confocal system (Perkin Elmer, Waltham, MA, USA) equipped with a fluorescence filter set for excitation at 488 nm. Only sperm cells showing evenly distributed fluorescence over the acrosomal region were considered acrosome-intact.

### 3.7. Statistical Analysis

Results are expressed as means ± SD. Comparisons were obtained with Student’s *t-*test for paired data and statistical significance was set at *p* < 0.05 (two-tailed). Relationships between pairs of variables were tested by least-squares linear regression. Pearson’s correlation coefficient *r* was used to quantify the strength of the relationships. The statistical significance of *r* was determined by ANOVA. All statistical analyses were performed with JMP^®^ 10 software (SAS Institute, Cary, NC, USA).

## 4. Conclusions

In this study, Asta is shown to ameliorate human sperm parameters, such as the Tyr-P pattern of the head and AR independently of the redox process of cells. This was achieved by incubating sperm in a commercial buffer (GB), which does not allow sperm to achieve the optimal endogenous ROS content for correct capacitation of a large number of cells. We have previously demonstrated that low ROS content, such as that obtained in the presence of ascorbic acid, is inconsistent with sperm functioning and survival [12]. Incubation in GB restricts the cells from generating the threshold of ROS to trigger capacitation in a high percentage of cells. In spite of this fact, Asta did manage to augment the percentage of ARC without increasing ROS levels. It is hypothesized that the addition of Asta bypasses the redox process involving ROS (H_2_O_2_) production and enables Tyr-phosphorylation of the sperm head protein. Asta can be inserted in the membrane and can therefore create a capacitation-like membrane alteration, allowing Tyr-P of the head. Once this has occurred, the following AR can take place and involve a higher number of cells. This hypothesis is further supported by the finding that a non-specific increase of the Tyr-P process obtained by inhibition of Tyr-protein phosphatases is insufficient to achieve correct capacitation.

## References

[1] Olds-Clarke P. (2003). Unresolved issues in mammalian fertilization. Int. Rev. Cytol..

[2] Liguori L., de Lamirande E., Minelli A., Gagnon C. (2005). Various protein kinases regulate human sperm acrosome reaction and the associated phosphorylation of Tyr residues and of the Thr-Glu-Tyr motif. Mol. Hum. Reprod..

[3] De Lamirande E., Leclerc P., Gagnon C. (1997). Capacitation as a regulatory event that primes spermatozoa for the acrosome reaction and fertilization. Mol. Hum. Reprod..

[4] Aitken R.J., Harkiss D., Knox W., Paterson M., Irvine D.S. (1998). A novel signal transduction cascade in capacitating human spermatozoa characterised by a redox-regulated, cAMP-mediated induction of tyrosine phosphorylation. J. Cell Sci..

[5] De Lamirande E., Gagnon C. (1993). A positive role for the superoxide anion in triggering hyperactivation and capacitation of human sperm. Int. J. Androl..

[6] Agarwal A., Sharma R.K., Nallella K.P., Thomas A.J., Alvarez J.G., Sikka S.C. (2006). Reactive oxygen species as an independent marker of male factor infertility. Fertil. Steril..

[7] Sharma R.K., Pasqualotto F.F., Nelson D.R., Thomas A.J., Agarwal A. (1999). The reactive oxygen species-total antioxidant capacity score is a new measure of oxidative stress to predict male infertility. Hum. Reprod..

[8] Moustafa M.H., Sharma R.K., Thornton J., Mascha E., Abdel-Hafez M.A., Thomas A.J., Agarwal A. (2004). Relationship between ROS production, apoptosis and DNA denaturation in spermatozoa from patients examined for infertility. Hum. Reprod..

[9] Agarwal A., Saleh R.A., Bedaiwy M.A. (2003). Role of reactive oxygen species in the pathophysiology of human reproduction. Fertil. Steril..

[10] Pasqualotto F.F., Sharma R.K., Nelson D.R., Thomas A.J., Agarwal A. (2000). Relationship between oxidative stress, semen characteristics, and clinical diagnosis in men undergoing infertility investigation. Fertil. Steril..

[11] Donà G., Fiore C., Tibaldi E., Frezzato F., Andrisani A., Ambrosini G., Fiorentin D., Armanini D., Bordin L., Clari G. (2011). Endogenous reactive oxygen species content and modulation of tyrosine phosphorylation during sperm capacitation. Int. J. Androl..

[12] Donà G., Fiore C., Andrisani A., Ambrosini G., Brunati A.M., Ragazzi E., Armanini D., Bordin L., Clari G. (2011). Evaluation of correct endogenous reactive oxygen species content for human sperm capacitation and involvement of the NADPH oxidase system. Hum. Reprod..

[13] Kobayashi M., Kakizono T., Nishio N., Nagai S., Kurimura Y., Tsuji Y. (1997). Antioxidant role of astaxanthin in the green alga Haematococcus pluvialis. Appl. Microbiol. Biotechnol..

[14] Terao J. (1989). Antioxidant activity of β-carotene-related carotenoids in solution. Lipids.

[15] Britton G. (1995). Structure and properties of carotenoids in relation to function. FASEB J..

[16] Kishimoto Y., Tani M., Uto-Kondo H., Iizuka M., Saita E., Sone H., Kurata H., Kondo K. (2010). Astaxanthin suppresses scavenger receptor expression and matrix metalloproteinase activity in macrophages. Eur. J. Nutr..

[17] Bennedsen M., Wang X., Willén R., Wadström T., Andersen L.P. (1999). Treatment of *H. pylori* infected mice with antioxidant astaxanthin reduces gastric inflammation, bacterial load and modulates cytokine release by splenocytes. Immunol. Lett..

[18] Comhaire F.H., El Garem Y., Mahmoud A., Eertmans F., Schoonjans F. (2005). Combined conventional/antioxidant “Astaxanthin” treatment for male infertility: A double blind, randomized trial. Asian J. Androl..

[19] Wolf A.M., Asoh S., Hiranuma H., Ohsawa I., Iio K., Satou A., Ishikura M., Ohta S. (2010). Astaxanthin protects mitochondrial redox state and functional integrity against oxidative stress. J. Nutr. Biochem..

[20] Pashkow F.J., Watumull D.G., Campbell C.L. (2008). Astaxanthin: A novel potential treatment for oxidative stress and inflammation in cardiovascular disease. Am. J. Cardiol..

[21] Bordin L., Ion-Popa F., Brunati A.M., Clari G., Low P.S. (2005). Effector-induced Syk-mediated phosphorylation in human erythrocytes. Biochim. Biophys. Acta.

[22] Nixon B., Mitchell L.A., Anderson A.L., McLaughlin E.A., O’bryan M.K., Aitken R.J. (2011). Proteomic and functional analysis of human sperm detergent resistant membranes. J. Cell. Physiol..

[23] Choi Y.H., Toyoda Y. (1998). Cyclodextrin removes cholesterol from mouse sperm and induces capacitation in a protein-free medium. Biol. Reprod..

[24] Botto L., Bernabò N., Palestini P., Barboni B. (2010). Bicarbonate induces membrane reorganization and CBR1 and TRPV1 endocannabinoid receptor migration in lipid microdomains in capacitating boar spermatozoa. J. Membr. Biol..

[25] Sleight S.B., Miranda P.V., Plaskett N.W., Maier B., Lysiak J., Scrable H., Herr J.C., Visconti P.E. (2005). Isolation and proteomic analysis of mouse sperm detergent-resistant membrane fractions: Evidence for dissociation of lipid rafts during capacitation. Biol. Reprod..

[26] World Health Organization (1999). WHO Laboratory Manual for the Examination of Human Semen and Sperm-Cervical Mucus Interaction.

[27] Saleh R.A., Agarwal A. (2002). Oxidative stress and male infertility: From research bench to clinical practice. J. Androl..

[28] Aitken R.J., Buckingham D.W., West K.M. (1992). Reactive oxygen species and human spermatozoa analysis of the cellular mechanisms involved in luminol- and lucigenin-dependent chemiluminescence. J. Cell. Physiol..

[29] Lukoseviciute K., Zilinskas H., Januskauskas A. (2004). Effect of exogenous progesterone on post-thaw capacitation and acrosome reaction of bovine sperm. Reprod. Domest. Anim..

